# Correlation between polymorphisms in microRNA-regulated genes and cervical cancer susceptibility in a Xinjiang Uygur population

**DOI:** 10.18632/oncotarget.15970

**Published:** 2017-03-07

**Authors:** Jing Fang, Ying Li, Jiayi Zhang, Mengdan Yan, Jingjie Li, Shan Bao, Tianbo Jin

**Affiliations:** ^1^ Department of Obstetrics and Gynecology, The First Affiliated Hospital of Xi'an Jiaotong University, Xi'an, Shaanxi 710061, China; ^2^ Department of Radiology, The First Affiliated Hospital of Xi'an Medical University, Xi'an, Shaanxi 710077, China; ^3^ Key Laboratory of Resource Biology and Biotechnology in Western China (Northwest University), Ministry of Education, School of Life Sciences, Northwest University, Xi’an, Shaanxi 710069, China; ^4^ Xi’an Tiangen Precision Medical Institute, Xi’an, Shaanxi 710075, China; ^5^ Clinic of Gynecology and Obstetrics, Hainan Provincial People's Hospital, Haikou 570102, China

**Keywords:** association study, cervical cancer, MicroRNA gene, single nucleotide polymorphisms (SNPs)

## Abstract

We explored the correlation between single nucleotide polymorphisms (SNPs) and susceptibility to cervical cancer (CC) in a Xinjiang Uygur population. Ten SNPs in eight miRNA-regulated genes were selected for analysis. Odds ratios (ORs) and 95% confidence intervals (95% CIs) were calculated using unconditional logistic regression analysis. Multivariate logistic regression analysis was used to detect correlations between SNPs and CC. We found that minor allele “C” of rs512715 in *NEAT1* was associated with an increased risk of CC in the allele, codominant, dominant, overdominant and log-additive models. Minor allele “C” of rs4777498 in *CELF6* was associated with an increased risk of CC in the recessive model. Minor allele “C” of rs3094 in *RNASE4* was associated with increased risk of CC in the allele, dominant and log-additive models. In clinical stage III/IV CC patients, minor allele “C” of rs3094 in *RNASE4* and minor allele “C” of rs8004334 in *JDP2* were associated with increased risk. In subtype squamous carcinoma CC patients, minor allele “C” of rs512715 in *NEAT1* and minor allele “C” of rs3094 in *RNASE4* were associated with increased risk. In subtype adenocarcinoma CC patients, minor allele “C” of rs3094 in *RNASE* was associated with increased risk.

## INTRODUCTION

Cervical cancer (CC) is the fourth most common malignancy in women, with 528,000 occurrences and 266,000 deaths in 2012 [1]. More than 85% of CC occurs in developing regions such as Eastern Africa, Melanesia, and Southern and Central Africa, where cervical CC accounts for more than 60% of gynecological cancers [2]. Cervical cancer is mainly attributable to human papillomaviruses (HPVs or PVs), which belong to the large Papillomaviridae family [1]. HPV particles include an approximately 8000-bp, double-stranded, closed circular DNA harboring eight genes [3].

Although previous studies have reported several biomarkers of CC, including p16^INK4a^ and Ki-67, few have investigated the relationship between CC risk and microRNA (miRNA)-regulated genes, including *CDK6*, *PTEN* and *NEAT1*, among others. MiRNAs are small (~19-25 nucleotides) non-coding RNA sequences that regulate gene expression through translational suppression accomplished through direct and/or triggered degradation of coding mRNAs mediated through binding to complementary sequences in the 3′ untranslated region (UTR) [4]. In this way, miRNAs play key roles in a variety of biological process, including cell apoptosis, proliferation, differentiation, development and tumorigenesis, which are involved in the pathogenesis of a variety of ailments, including cancer, nephropathy and renovascular disease [5–7]. Consequently, how miRNA-regulated genes affect CC risk would seem to be a potentially meaningful investigation. We therefore investigated the relationships between single nucleotide polymorphisms (SNPs) in miRNA-regulated genes and the risk of CC. In the present case-control study, we selected 10 SNPs in eight miRNA-regulated genes and performed a comprehensive association analysis in a Xinjiang Uygur population.

## RESULTS

The basic information on the 10 SNPs examined in this study is summarized in Table 1. We found that rs2285332 (*p* = 0.008), rs11202607 (*p* = 0.022) and rs680413 (*p* = 0.013) deviated from the Hardy-Weinberg Equilibrium (*p <* 0.05), and were excluded from our analysis. We found two SNPs were significantly associated with CC (rs512715, *NEAT1*, OR = 1.354, 95% CI: 1.039-1.764, *p* = 0.025; rs3094, *RNASE4*, OR = 1.359, 95% CI: 1.051-1.758, *p* = 0.019).

**Table 1:** Basic information on the SNPs examined in this study

SNPs	MiRNA	Gene	Chr	Band	Role	Alleles	MAF		HWE *p*	OR	95% CI		*p*
							casae	control					
rs2285332	hsa-miR-218	CDK6	chr7	7q21.2	3′ UTR	C/G	0.504	0.437	0.008	1.310	1.029	1.669	0.028
rs701848	hsa-miR-23b	PTEN	chr10	10q23.31	3′ UTR	T/C	0.536	0.489	0.192	1.207	0.948	1.536	0.126
rs11202607	hsa-miR-23b	PTEN	chr10	10q23.31	3′ UTR	T/C	0.079	0.065	0.022	1.235	0.774	1.969	0.375
rs680413	hsa-mir-342-3p	NEAT1	chr11	11q13.1	5′ UTR	G/T	0.130	0.128	0.013	1.013	0.707	1.452	0.943
rs512715	hsa-mir-342-3p	NEAT1	chr11	11q13.1	5′ UTR	C/G	0.326	0.263	0.221	1.354	1.039	1.764	0.025
rs1133822	hsa-mir-342-3p	LOC105372481	chr19	19q13.43	-	G/A	0.302	0.328	1.000	0.885	0.682	1.147	0.355
rs4777498	hsa-mir-375	CELF6	chr15	15q23	3′ UTR	C/A	0.202	0.184	0.693	1.124	0.829	1.525	0.452
rs3094	hsa-mir-590-5p	RNASE4	chr14	14q11.2	3′ UTR	C/T	0.360	0.293	0.476	1.359	1.051	1.758	0.019
rs8004334	hsa-mir-590-5p	JDP2	chr14	14q24.3	Promoter	C/T	0.435	0.379	0.615	1.263	0.988	1.614	0.062
rs3733839	hsa-mir-590-5p	LOC153684	chr5	5p12	5′ UTR	C/G	0.265	0.273	0.293	0.961	0.732	1.262	0.774

Abbreviations: SNPs: Single nucleotide polymorphisms; MiRNA: microRNA; Chr: chromosome; MAF: Minor allele frequency; HWE: Hardy-Weinberg equilibrium; OR: Odds ratio; CI: Confidence interval.

*p*-values were calculated using Pearson's χ^2^ test. Values of *p* < 0.05 were considered statistically significant.

We then conducted an unconditional logistic regression analysis, and the positive results are illustrated in Table 2. We found three SNPs that were associated with increased CC risk in different models. The minor allele “C” of rs512715 increased CC risk in the codominant (OR = 1.56, 95% CI: 1.09-2.24, *p* = 0.044), dominant (OR = 1.55, 95% CI: 1.10-2.18, *p* = 0.012), overdominant (OR = 1.46, 95% CI: 1.03-2.08, *p* = 0.032) and log-additive (OR = 1.34, 95% CI: 1.03-1.75, *p* = 0.027) models. The minor allele “C” of rs4777498 increased CC risk in the recessive model (OR = 2.40, 95% CI: 1.01-5.70, *p* = 0.041). And the minor allele “C” of rs3094 increased CC risk in dominant (OR = 1.47, 95% CI: 1.04-2.08, *p* = 0.027) and log-additive (OR = 1.35, 95% CI: 1.04-1.74, *p* = 0.021) models.

**Table 2:** Unconditional logistic regression analysis of the association between SNPs and CC risk

SNPs	Model	Genotype	Controls n (%)	Cases n (%)	OR (95% CI)	*p*	AIC	BIC
rs512715	Codominant	G/G	159 (55.8%)	111 (44.9%)	1	0.044	734.5	747.4
		C/G	102 (35.8%)	111 (44.9%)	1.56 (1.09-2.24)			
		C/C	24 (8.4%)	25 (10.1%)	1.49 (0.81-2.75)			
	Dominant	G/G	159 (55.8%)	111 (44.9%)	1	0.012	732.5	741.1
		C/G-C/C	126 (44.2%)	136 (55.1%)	1.55 (1.10-2.18)			
	Recessive	G/G-C/G	261 (91.6%)	222 (89.9%)	1	0.5	738.3	746.9
		C/C	24 (8.4%)	25 (10.1%)	1.22 (0.68-2.20)			
	Overdominant	G/G-C/C	183 (64.2%)	136 (55.1%)	1	0.032	734.2	742.7
		C/G	102 (35.8%)	111 (44.9%)	1.46 (1.03-2.08)			
	Log-additive	---	---	---	1.34 (1.03-1.75)	0.027	733.9	742.5
rs4777498	Codominant	A/A	188 (66%)	163 (66%)	1	0.1	736.2	749
		C/A	89 (31.2%)	68 (27.5%)	0.88 (0.60-1.29)			
		C/C	8 (2.8%)	16 (6.5%)	2.31 (0.96-5.53)			
	Dominant	A/A	188 (66%)	163 (66%)	1	0.99	738.8	747.3
		C/A-C/C	97 (34%)	84 (34%)	1.00 (0.70-1.43)			
	Recessive	A/A-C/A	277 (97.2%)	231 (93.5%)	1	0.041	734.6	743.2
		C/C	8 (2.8%)	16 (6.5%)	2.40 (1.01-5.70)			
	Overdominant	A/A-C/C	196 (68.8%)	179 (72.5%)	1	0.35	737.9	746.5
		C/A	89 (31.2%)	68 (27.5%)	0.84 (0.57-1.22)			
	Log-additive	---	---	---	1.12 (0.83-1.51)	0.46	738.3	746.8
rs3094	Codominant	T/T	145 (50.9%)	102 (41.3%)	1	0.067	735.4	748.2
		T/C	113 (39.6%)	112 (45.3%)	1.41 (0.98-2.03)			
		C/C	27 (9.5%)	33 (13.4%)	1.74 (0.98-3.07)			
	Dominant	T/T	145 (50.9%)	102 (41.3%)	1	0.027	733.9	742.5
		T/C-C/C	140 (49.1%)	145 (58.7%)	1.47 (1.04-2.08)			
	Recessive	T/T-T/C	258 (90.5%)	214 (86.6%)	1	0.16	736.8	745.4
		C/C	27 (9.5%)	33 (13.4%)	1.47 (0.86-2.53)			
	Overdominant	T/T-C/C	172 (60.4%)	135 (54.7%)	1	0.18	737	745.6
		T/C	113 (39.6%)	112 (45.3%)	1.26 (0.89-1.78)			
	Log-additive	---	---	---	1.35 (1.04-1.74)	0.021	733.5	742

Abbreviations: SNPs: Single nucleotide polymorphisms; OR: Odds ratio; CI: Confidence interval.

*p-*values were calculated using Pearson's χ^2^ test. Values of *p* < 0.05 were considered statistically significant.

The associations between SNPs and different clinical stages and CC subtypes were assessed, and the positive results are illustrated in Table 3. In clinical stage III/IV patients, we found rs3094 (OR = 1.51, 95% CI: 1.06-2.14, *p* = 0.021) and rs8004334 (OR = 1.60, 95% CI: 1.15-2.24, *p* = 0.006) to be associated with an increased CC risk. In subtype squamous carcinoma patients, we found rs512715 (OR = 1.37, 95% CI: 1.05-1.79, *p* = 0.021) and rs3094 (OR = 1.31, 95% CI: 1.01-1.70, *p* = 0.043) to be associated with an increased CC risk. And in subtype adenocarcinoma patients, we found rs3094 (OR = 4.02, 95% CI: 1.11-11.24, *p* = 0.004) to be associated with an increased CC risk.

**Table 3:** Association between SNPs and different clinical CC subtypes

SNPs	Clinical Stages				Subtypes			
	I-II		III-IV		squamous carcinoma		adenocarcinoma	
	OR (95%CI)	p	OR (95%CI)	p	OR (95%CI)	p	OR (95%CI)	p
rs512715	1.23 (0.90-1.68)	0.187	1.41 (0.98-2.02)	0.060	1.37(1.05-1.79)	0.021	0.93 (0.30-2.94)	0.906
rs3094	1.26 (0.93-1.70)	0.136	1.51 (1.06-2.14)	0.021	1.31(1.01-1.70)	0.043	4.02 (1.44-11.24)	0.004
rs8004334	1.07 (0.81-1.43)	0.624	1.60 (1.15-2.24)	0.006	1.27(0.99-1.63)	0.056	0.98 (0.35-2.74)	0.974

Abbreviations: SNPs: Single nucleotide polymorphisms; OR: Odds ratio; CI: Confidence interval.

*p-*values were calculated using Pearson's χ^2^ test. Values of *p* < 0.05 were considered statistically significant.

## DISCUSSION

In the present study, we found that four SNPs belonging to four miRNA-regulated genes were associated with CC risk. These were rs512715 in *NEAT1* regulated by hsa-mir-342-3p, rs4777498 in *CELF6* regulated by hsa-mir-375, and rs3094 in *RNASE4* and rs8004334 in *JDP2*, both regulated by hsa-mir-590-5p.

In humans, miRNAs are transcribed by RNA polymerase II in the nucleus as pri-miRNAs, which may contain two or more mature miRNAs. Subsequently, pri-miRNAs are processed by RNase III to form pre-miRNAs exported to the cytosol, carried by exportin 5, after which the pre-miRNAs are processed by Dicer in the cytosol to mature miRNAs. One strand of the mature miRNA is then incorporated with RNA-induced silencing complex (RISC), directing it to target mRNA [8].

The minor allele “C” of rs512715 increased CC risk in the allele, codominant, dominant, overdominant and log-additive models. Rs512715 belongs to *NEAT1*, which is regulated by hsa-mir-342-3p. We know of no other study relating *NEAT1* to CC risk, though a Chinese study found a relationship between *NEAT1* and bladder cancer [9, 10]. In addition, an American study found hsa-mir-342-3p to be related to irritable bowel syndrome [11]. In a German study, significant upregulation of hsa-miR-342-3p was detected in the brains of macaques infected with bovine spongiform encephalopathy, and in a pilot study they also showed that hsa-miR-342-3p was upregulated in brain samples from humans with type 1 or type 2 sporadic Creutzfeldt-Jakob disease [12]. We have so far detected no direct evidence of a specific relationship between hsa-miR-342-3p and CC, and we suggest that this miRNA likely plays a general role in the regulation of multiple target genes in disease. However, the detailed mechanism by which hsa-miR-342-3p exerts gene effects in CC deserves further investigation.

The minor allele “C” of rs4777498 increased CC risk in the recessive model. Rs4777498 belongs to *CELF6*, which is regulated by hsa-mir-375. An American study found that *CELF6* is highly expressed in diencephalic nuclei and neuromodulatory cell populations of the mouse brain [13]. Previous studies also reported hsa-mir-375 to be related to pancreatic cancer and early stage breast cancer [14, 15]. In breast cancer, higher levels of hsa-mir-375 were expressed in ER-α-positive than ER-α-negative or normal cells, which led to the suggestion that hsa-miR-375 up-regulation is a key driver of cell proliferation and an early event in tumorigenesis in ER-α-positive tissues [16]. However, a detailed understanding of the mechanism by which hsa-mir-375 affects CC risks will require further investigation.

The minor allele “C” of rs3094 increased CC risk in the allele, dominant and log-additive models. In clinical stage III/IV patients, the minor allele “C” of rs3094 and minor allele “C” of rs8004334 were associated with increased CC risk. Rs3094 belongs to *RNASE4* while rs8004334 belong to *JDP2*, and both are regulated by hsa-mir-590-5p. Previous studies showed *RNASE4* to be associated with high-altitude adaptation, metabolic syndrome and neuron degeneration [17–19], while *JDP2* was associated with heart failure [20]. Hsa-mir-590-5p is reportedly related to cardiac differentiation through down-regulation of TGFB signaling [21]. TGFB1-induced activation of Smad 2, -3, -4 leads to direct inhibition of STAT5 transactivation and STAT5-mediated transcription of downstream target genes, including miR-590 [22]. TGFB1 inhibits STAT5 expression at the protein level with no effect on mRNA expression. Whether there is a relationship between the mechanism of hsa-mir-590-5p-mediated effects on CC risk and TGFB signaling warrants further investigation.

There are two intrinsic limitations to this study. 1) The sample size was not large enough to obtain illative combinatory associations between SNPs and CC. 2) Selection bias may be unavoidable since this was a hospital-based study. Therefore, larger well-designed studies combined with CC classification are needed to confirm the observed associations and clarify the potential biological mechanisms of these SNPs in CC.

In summary, we have identified significant associations between rs512715 (*NEAT1*), rs4777498 (*CELF6*), rs3094 (*RNASE*) and rs8004334 *(JDP2*) and CC risk in Xinjiang Uygur population.

## MATERIALS AND METHODS

### Study participants

A total of 532 subjects, including 247 patients with cervical cancer and 285 healthy women were recruited at the People's Hospital of Xinjiang Uyghur Autonomous Region between January 2014 and Jun 2016. The included patients were recently diagnosed with primary CC based on cervical biopsy with histopathological confirmation. We excluded patients with other cancers who underwent radiotherapy or chemotherapy. Controls were healthy, unrelated individuals selected randomly from the medical examination center of the hospital. All participants were women at least 18 years old in good mental condition who had at least three generations of paternal ancestry in their ethnicity (Xinjiang Uygur population). Tumors were staged according to International Federation of Gynecology and Obstetrics (FIGO) classification. Informed consent was obtained from all participants, and the study protocols were approved by the institutional review board of the People's Hospital of Xinjiang Uyghur Autonomous Region.

### SNP selection and genotyping

Candidate SNPs were selected from among previously published polymorphisms associated with CC. Validated SNPs were selected with a MAF > 5% in the HapMap Asian population [23]. Venous blood samples (5 ml) were collected from each patient during laboratory examination. Genomic DNA was extracted from whole blood samples using a Gold Mag-Mini Whole Blood Genomic DNA Purification Kit (version 3.0; TaKaRa, Japan) [24] and stored at -80°C after centrifugation. DNA concentrations were evaluated using spectrometry (DU530 UV/VIS spectrophotometer, Beckman Instruments, Fullerton, CA, USA). We used Sequenom MassARRAY Assay Design 3.0 Software to design the Multiplexed SNP MassEXTEND assays [25]. SNP genotyping was done with a Sequenom MassARRAY RS1000 using the standard protocol recommended by the manufacturer [25]. The primer sequences used for genotyping are listed in Table 4. Data management and analyses were performed using Sequenom Typer 4.0 software as previously described [25, 26].

**Table 4:** Primers used for this study

SNP_ID	1st-PCRP	2nd-PCRP	UEP_SEQ
rs2285332	ACGTTGGATGTGAGCTGC TTCAGTGTAACC	ACGTTGGATGCTTTG CCAAAAGCTAAGCAG	gGCCAAAAGCTAAGCAGTGGTGAA
rs701848	ACGTTGGATGATAGTGCTC CCCCGAGTTG	ACGTTGGATGCTCCG CTTAAAATCGTATGC	TGATTTTTTTTAAGAAGTGAAATTGA
rs11202607	ACGTTGGATGTATTTATG ACCTGGCCCTCC	ACGTTGGATGTTACAA TTTCGGGCACCGCA	cTTCGGGCACCGCATATTAAAA
rs680413	ACGTTGGATGCCTAGA CCTAGTCTCCTTGC	ACGTTGGATGGGGAG AGATGACTGAGTTAG	ggTGACTGAGTTAGATGAGAC
rs512715	ACGTTGGATGAACAG CCACTCGGCTTACTG	ACGTTGGATGCCCTT CTTCCTCCCTTTAAC	AACTTATCCATTCACTTAAAACATTA
rs1133822	ACGTTGGATGCCTTC GTTCTCCTTCGTTTG	ACGTTGGATGTTTC TCTGCTCTGGCAGACC	gGGGCACCACTTGTCACGG
rs4777498	ACGTTGGATGGGATTG TGGATTGTGGGTTC	ACGTTGGATGTGAG GTCTAGGCTCACATGC	GCTCACATGCAGGTAAT
rs3094	ACGTTGGATGGATTATC GCGAGTGGTTGAC	ACGTTGGATGAATGAG CTGAGGAGACAGAG	ccGCTGAGGAGACAGAGCCTGGG
rs8004334	ACGTTGGATGACTAAA GGCCTCCCAAGTCA	ACGTTGGATGTCCTA CTGGGCCTTTGCTTC	aTTTGCTTCCCCCACAAATTAAAT
rs3733839	ACGTTGGATGCCATGC AACCAATTCCATCC	ACGTTGGATGGTCTCC TGACTTGTCAAGGC	TCCTCTGCACCTGTCCT

### Statistical analysis

Statistical analyses were performed using Microsoft Excel (Redmond, WA, USA) and the SPSS 17.0 statistical package (SPSS, Chicago, IL, USA). All *p* values in this study were two-sided, and *p* ≤ 0.05 after Bonferroni correction was considered the statistical significance threshold [27]. An exact test was used to assess the departure of each SNP frequency from Hardy-Weinberg equilibrium (HWE) in the controls. We compared allele frequencies between cases and controls using the χ^2^ test. To assess the association of single SNPs with the risk of CC, five genetic models (codominant, dominant, recessive, over-dominant and log-additive) were applied using PLINK software (http://www.cog-genomics.org/plink2/). Odds ratios (ORs), 95% confidence intervals (95% CIs), and *p* values were calculated using unconditional logistic regression analysis [28–30].
